# Commentary: Post-translational modifications in retinoblastoma: mechanisms, immune regulation, and therapeutic opportunities

**DOI:** 10.3389/fimmu.2026.1881594

**Published:** 2026-07-15

**Authors:** Lidetian Hu, Haixing Cao, Yujie Zhang, Xiang Ma

**Affiliations:** Department of Ophthalmology, The First Affiliated Hospital of Dalian Medical University, Dalian, China

**Keywords:** biomarker validation, immunotherapy, ocular drug delivery, ocular safety, post-translational modification, retinoblastoma, translational oncology

## 1 Introduction

Xu and colleagues provide a timely and wide-ranging review of post-translational modifications (PTMs) in retinoblastoma, linking RB-centered signaling, chromatin regulation, metabolic adaptation, therapeutic resistance, and emerging immunotherapeutic opportunities (1). This synthesis is valuable because retinoblastoma research increasingly requires mechanism-based strategies that preserve not only life, but also the globe and vision. The key challenge, however, is not whether PTMs are biologically relevant in retinoblastoma, but whether PTM-associated observations can be prioritized into clinically testable dependencies. Several conceptual and methodological boundaries therefore require sharper definition before PTM biology can be interpreted as a translational roadmap.

## 2 Target specificity and evidence hierarchy

A major issue is that heterogeneous evidence streams are often integrated into a common therapeutic narrative. Human tumor observations, orthotopic models, conventional xenografts, established cell lines, and mechanistic studies from non-retinoblastoma systems do not provide equivalent support for clinical prioritization. For example, in Xu et al. usefully summarizes numerous compounds reported to modulate PTM-related pathways in retinoblastoma; however, many entries are supported primarily by established cell-line systems such as Y79 or WERI-Rb1, with limited orthogonal validation in human tumor or patient-derived models. A PTM axis inferred from a non-retinal cancer model, a phosphorylation change in standard cell lines, and a dependency reproduced in patient-derived or orthotopic retinoblastoma systems should carry different inferential weight. Preclinical oncology has repeatedly shown that insufficiently validated experimental findings can impede rather than accelerate clinical translation (2). Without such evidence, PTM observations from non-retinal models or standard cell lines should be explicitly labeled as hypothesis-generating rather than validated retinoblastoma dependencies.

A related concern is the definition of “PTM-directed therapy.” Not every compound that changes phosphorylation, acetylation, ubiquitination, glycosylation, or metabolic readouts should be classified as a PTM-targeting intervention. Some agents directly modulate PTM writers, erasers, readers, or substrate-specific modification events. Others alter PTM markers indirectly through oxidative stress, apoptosis, mitochondrial dysfunction, growth-factor signaling, or nonspecific pathway inhibition. For instance, HDAC inhibitors more directly engage acetylation machinery, whereas 2-deoxy-D-glucose may alter glycosylation-related or metabolic stress readouts without necessarily constituting a substrate-specific PTM intervention. This distinction is not semantic. A secondary decrease in phosphorylated AKT or ERK does not establish a PTM-specific therapeutic mechanism. Future reviews should separate direct PTM-enzyme targeting, substrate-selective PTM modulation, PTM-associated pathway inhibition, and nonspecific cytotoxic effects. Most PTM enzymes modify multiple substrates, making therapeutic-index optimization particularly challenging in a pediatric intraocular tumor. Broader PTM drug-discovery literature similarly emphasizes that feasibility depends not only on biochemical modulation, but also on target selectivity, substrate context, delivery, and safety (3).

## 3 Clinical translation, ocular safety, and biomarker validation

This issue is particularly important in retinoblastoma. Unlike many systemic cancers, retinoblastoma therapy must be judged through the combined endpoints of survival, globe salvage, visual preservation, ocular toxicity, developmental safety, and feasibility of local drug delivery. A PTM regulator may be chemically tractable yet clinically implausible if tumor-selective dependence, intraocular pharmacokinetics, retinal safety, or compatibility with existing treatment routes is not established.

Targeted protein degraders, including PROTACs, molecular glues, and related ubiquitin-proteasome–directed platforms, represent a further extension of ubiquitination-centered PTM intervention. In retinoblastoma, however, their translational relevance should be assessed through an ocular-delivery and safety framework. PROTACs and some degrader platforms may face challenges related to molecular size, permeability, formulation, and tissue exposure, whereas molecular glues may be more drug-like but still require confirmation of adequate intraocular exposure, tumor engagement, and retinal safety (4–6). This issue is particularly relevant because posterior-segment drug delivery is constrained by anatomical and physiological ocular barriers, including the blood-retinal barrier (6). Because ubiquitin-proteasome pathways also regulate normal retinal, neuronal, and developing tissues, degrader-based PTM strategies should be prioritized only when tumor-selective dependency, ocular pharmacokinetic feasibility, compatibility with local delivery routes, and acceptable toxicity profiles have been demonstrated.

Contemporary retinoblastoma treatment reviews emphasize that current therapeutic progress is closely linked to eye-directed approaches such as ophthalmic artery chemotherapy and intravitreal chemotherapy, whereas novel drugs, oncolytic viruses, and immunotherapy remain emerging rather than established components of routine care (7). Moreover, a recent systematic review of intravitreal chemotherapy identified retinal toxicity, cataract, and vitreous hemorrhage among the most common ocular complications, reinforcing the need to evaluate local safety alongside antitumor activity (8). A minimal translational score should assign credit for human tumor PTM validation, genetic or pharmacologic rescue in retinoblastoma-relevant models, ocular pharmacokinetic feasibility, and retinoblastoma-specific immune or biomarker endpoints.

The PTM–immunotherapy interface requires a similarly retinoblastoma-specific validation pathway. Evidence from RB1 biology, immune checkpoint regulation, or PTM-mediated immune modulation in other malignancies can generate important hypotheses, but it cannot by itself establish immune targetability in pediatric intraocular retinoblastoma. Antigen density, intratumoral heterogeneity, immune-cell infiltration, prior chemotherapy exposure, differentiation status, and metastatic context may substantially modify therapeutic relevance. Recent retinoblastoma data suggest that B7-H3 and GD2 are promising immunotherapeutic targets, while also underscoring variability in antigen expression across tumor samples (9). PTM–immunotherapy combinations should therefore be evaluated using measurable retinoblastoma-specific endpoints, including antigen density, immune recruitment, ocular inflammatory toxicity, antigen loss, and resistance evolution.

Biomarker strategy is the practical bridge between mechanistic hypotheses and patient-level translation. Direct tumor biopsy is generally avoided in intraocular retinoblastoma, making aqueous humor liquid biopsy particularly relevant for molecular monitoring. Prospective work has shown that aqueous humor can support diagnostic, prognostic, and therapeutic biomarker development in retinoblastoma (10). Notably, Xu et al. themselves acknowledge that some PTM classes, such as SUMOylation in retinoblastoma, remain essentially unreported, and that lactylation warrants further investigation. This reinforces rather than weakens the need for a graded translational framework. Integrating PTM-related biomarkers with aqueous humor analyses, antigen profiling, and longitudinal treatment response may provide a more realistic translational route than relying on static tissue, cell-line, or cross-cancer evidence alone.

## 4 Conclusion

The broader lesson from this review is that translational oncology frameworks should separate discovered biology, validated dependency, biomarker-ready mechanism, and clinically plausible intervention. This distinction is especially important in retinoblastoma, where experimental enthusiasm must be balanced against the pediatric, intraocular, and vision-preserving context of care. Incorporating evidence hierarchy, a stricter definition of PTM-directed therapy, retinoblastoma-specific immune validation, ocular safety assessment, and longitudinal biomarker strategies would strengthen the field. These refinements would not diminish the value of PTM research; rather, they would help convert a compelling molecular catalog into a prioritized, testable, and clinically disciplined translational agenda. The next step for the field is not a longer PTM catalog, but a ranked translational map linking mechanism, biomarker, delivery route, and clinically meaningful endpoints.

## References

[1] Xu L Wang B Li W Luo X Wang L Zheng Y . Post-translational modifications in retinoblastoma: mechanisms, immune regulation, and therapeutic opportunities. Front Immunol. (2026) 17:1820785. doi: 10.3389/fimmu.2026.1820785 42212122 PMC13212488

[2] Begley CG Ellis LM . Drug development: raise standards for preclinical cancer research. Nature. (2012) 483:531–3. doi: 10.1038/483531a 22460880

[3] Cao Y Yu T Zhu Z Zhang Y Sun S Li N . Exploring the landscape of post-translational modification in drug discovery. Pharmacol Ther. (2025) 265:108749. doi: 10.1016/j.pharmthera.2024.108749 39557344

[4] Edmondson SD Yang B Fallan C . Proteolysis targeting chimeras (PROTACs) in "beyond rule-of-five" chemical space: recent progress and future challenges. Bioorg Med Chem Lett. (2019) 29:1555–64. doi: 10.1016/j.bmcl.2019.04.030 31047748

[5] Dong G Ding Y He S Sheng C . Molecular glues for targeted protein degradation: from serendipity to rational discovery. J Med Chem. (2021) 64:10606–20. doi: 10.1021/acs.jmedchem.1c00895 34319094

[6] Thrimawithana TR Young S Bunt CR Green C Alany RG . Drug delivery to the posterior segment of the eye. Drug Discov Today. (2011) 16:270–7. doi: 10.1016/j.drudis.2010.12.004 21167306

[7] Schaiquevich P Francis JH Cancela MB Carcaboso AM Chantada GL Abramson DH . Treatment of retinoblastoma: what is the latest and what is the future. Front Oncol. (2022) 12:822330. doi: 10.3389/fonc.2022.822330 35433448 PMC9010858

[8] Lavasidis G Strongylis M Tzamalis A Tsinopoulos I Ntzani EE . Safety of intravitreal chemotherapy in the management of retinoblastoma: a systematic review of the literature. Crit Rev Oncol Hematol. (2024) 200:104423. doi: 10.1016/j.critrevonc.2024.104423 38897313

[9] Sujjitjoon J Ng WL Kongkla K Jarutatsanangkoon K Chongsutakawewong K Subhadhirasakul S . B7-H3 and GD2 overexpression as immunotherapeutic targets in retinoblastoma. Sci Rep. (2026) 16:4210. doi: 10.1038/s41598-025-34331-6 41521230 PMC12859113

[10] Berry JL Pike S Shah R Reid MW Peng CC Wang Y . Aqueous humor liquid biopsy as a companion diagnostic for retinoblastoma: implications for diagnosis, prognosis, and therapeutic options: five years of progress. Am J Ophthalmol. (2024) 263:188–205. doi: 10.1016/j.ajo.2023.11.020 38040321 PMC11148850

